# 

**Published:** 1905-05-27

**Authors:** 


**NOTICE TO CORRESPONDENTS.**

All MSS., Letters, Books for Review, and other matters intended for the Editor should be addressed The Editor, "The Hospital," The Hospital Buildings, 28 & 29 Southampton Street, Strand, W.C.

The Editor cannot undertake to return rejected MS., even when accompanied by stamped directed envelope.

**Notice to Advertisers and Subscribers.**

All Advertisements, Orders for Copies of The Hospital, and Business Communications should be addressed to **THE MANAGER (Not to the Editor),** The Hospital, 28 & 29 Southampton Street, Strand, London, W.C.

The Cost of Subscription, post free, is at follows:—Per week, 3½d.; per quarter, 3s. 3d.; per half-year, 6s. 6d.; per annum, 13s. Foreign and Colonial:—Per annum, 19s. 6d., payable, in all cases, in advance.

**NOTICE.—Vol. XXXVII. of "The Hospital" October 1904 to March 1905), in handsome cloth binding, will shortly be on sale at the office of this Journal, price 10s. 6d. Cases for binding the Numbers contained in this Volume 2s. Index to Vol. XXXVII., 3½d., post free.**

**The Monthly Part for May is now ready. Price 1s., by post, 1s. 3d.**

### Medical Appointments.

**E. Frank R. Alford**, M.R.C.S., L.R.C.P.Lond., House Physician at the Paddington Green Children's Hospital, W. **James D. C. Allen**, M.B., C.M.Edin., Public Vaccinator and Medical Officer to the Binfield District of the Easthampstead Union. **John Anderson**, M.D.Aberd., Officer of Health for the East Riding of the Council Shire of Ballan, Victoria, Australia. **John C. Baird**, M.B., Ch.B.Melb., Officer of Health for the Shire of Healesville, Victoria, Australia. **W. H. Beaumont**, M.B., B.S.Cantab., Certifying Surgeon under the Factory and Workshop Act for the Sedbergh District of the County of York. **W. Foster Cross**, M.R.C.S.Eng., L.R.C.P.Lond., an additional Non-Resident Anæsthetist at St. Bartholomew's Hospital. **Walter Gordon Gray**, L.R.C.P., L.M.Edin., M.R.C.S., Medical Officer of Health for the Holsworthy (Devon) Urban District Council. **John Harris**, M.D.Aberd., Honorary Assistant Physician to the Sydney Hospital, New South Wales, Australia. **T. C. Lawson**, M.R.C.S., L.S.A., Certifying Surgeon under the Factory and Workshop Act for the Stokenchurch District of the county of Buckingham. **John Lewis Lock**, M.A., M.B., B.C.Cantab., M.R.C.S., L.R.C.P., Medical Officer of Health of the Uxbridge Urban District. **Charles Crawfurd Macknight**, M.B.&Ch.M.Edin., Officer of Health for the Shire of Warrnambool, Victoria, Australia. **W. E. Manby**, M.B.Cantab., Certifying Surgeon under the Factory and Workshop Act for the Bridport District of the county of Dorset. **Robert Saundby**, M.D.Edin., F.R.C.P.Lond., Consulting Physician to the West Bromwich District Hospital. **Thomas Walker Sinc'air**, M.D.Melb., on probation for six months as Assistant Medical Officer of Health for the Metropolitan Combined Districts, New South Wales, Australia. **E. J. Smyth**, M.D., B.S.Lond., Honorary Ophthalmic Surgeon to the Royal Surrey County Hospital, Guildford. **George Henry Trenfie d**, L.R.C.P.Lond., M.R.C.S., Medical Officer to the Almshouses under the management of the trustees of the Bristol Municipal Charities. **W. L. Watkins**, L.K.Q.C.P.Irel., Medical Superintendent at Yarra Bend Asylum, Victoria, Australia.

### A HANDBOOK FOR NURSES.
By J. K. WATSON, M.D.
NEW EDITION. With Particulars of the Care of the Dead, and Examination Questions based upon Chapters.
5/4 post free.

### THE HUMAN BODY.
**A Manual of Physiology and Personal Hygiene.**
By B. P. COLTON. Profusely Illustrated.

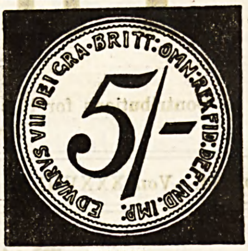

EACH.

### ELEMENTARY TREATISE ON THE LIGHT TREATMENT FOR NURSES.
By JAMES H. SEQUEIRA, M.D. Illustrated. 2/9 post free.

### ELEMENTARY ANATOMY AND SURGERY FOR NURSES.
By Dr. McADAM ECCLES.

### NURSING IN DISEASES OF THE THROAT, NOSE AND EAR.
By Dr. P. MACLEOD YEARSLEY.

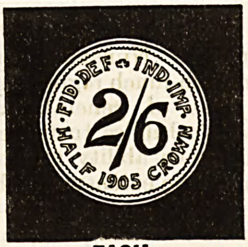

EACH.

### ELEMENTARY PHYSIOLOGY FOR NURSES.
By Dr. C. F. MARSHALL. Illustrated.

### ELEMENTS OF ANATOMY AND PHYSIOLOGY.
By BERNARD SECRETAN. Illustrated. 2/2 post free.

### PRACTICAL GUIDE TO SURGICAL BANDAGING AND DRESSINGS.
By Dr. JOHNSON SMITH.

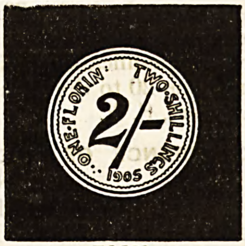

EACH.

### HOW TO BECOME A CERTIFIED MIDWIFE.
By Dr. APPEL. 1/1 post free.

### MODERN NURSING OF CONSUMPTION.
By Dr. JANE WALKER. 1/2 post free.

### MIDWIVES' POCKET-BOOK.
By HONNOR MORTEN.

### FEVERS AND INFECTIOUS DISEASES:
**Their Nursing and Practical Management.**
By Dr. HARDING.

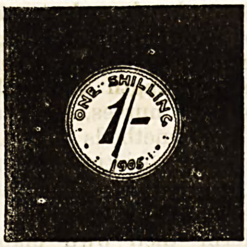

EACH.

### THE SCHOTT TREATMENT FOR CHRONIC HEART DISEASE.
By RICHARD GREENE, F.R.C.P. Ed.

### POCKET-BOOK OF TEMPERATURE CHARTS.
Containing 17 M. and E. Charts and 8 Four-hour Charts.
7d. post free.

### PREPARATION FOR OPERATION IN PRIVATE HOUSES.
By A SURGEON.

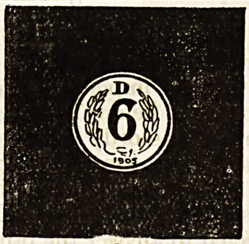

EACH.

NEW CATALOGUE OF NURSING MANUALS Free on Application.

THE SCIENTIFIC PRESS, LTD.,
Publishers of Nursing Text-books, Case-books, and Charts of all descriptions,
28 & 29 Southampton Street, Strand, London, W.C.

### The NURSING MIRROR.

**The Nurse's Own Paper.**

**Price ONE PENNY Weekly.**

Of all Booksellers and Newsagents, or posted direct from the Office.

*Write for Subscription Rates to The Manager,*

THE NURSING MIRROR, The Hospital Building, 28 & 29 Southampton Street, Strand, London, W.C.

